# The Balance Between the Natriuretic Peptides and the Renin-Angiotensin-Aldosterone System in the Preservation of Ideal Cardiovascular Health

**DOI:** 10.3390/jcm14020626

**Published:** 2025-01-19

**Authors:** Speranza Rubattu, Giovanna Gallo, Massimo Volpe

**Affiliations:** 1 Clinical and Molecular Medicine Department, School of Medicine and Psychology, Sapienza University of Rome, 00189 Roma, Italy; giovanna.gallo@uniroma1.it (G.G.); massimo.volpe@uniroma1.it (M.V.); 2 IRCCS Neuromed, 86077 Pozzilli, Italy; 3 Cardiology Unit, Sant’Andrea University Hospital, 00189 Rome, Italy; 4 IRCCS S.Raffaele, 00163 Rome, Italy

**Keywords:** cardiovascular health, natriuretic peptides, RAAS, Ang (1-7), primary prevention

## Abstract

A healthy lifestyle plays a key role for maintaining the cardiovascular health (CVH) status and prevent cardiovascular disease occurrence. In fact, a healthy lifestyle was included in the AHA Cardiovascular Health score (Life’s Simple 7 [LS7]), subsequently updated to Life’s Simple 8 [LS8]. Apart from the importance of controlling conventional cardiovascular risk factors, increasing evidence supports the contributory role of cardiovascular hormones. Higher levels of natriuretic peptides (NPs) and lower levels of renin and aldosterone were significantly associated to CVH. NT-proBNP levels showed a direct relationship with CVH scores in large general Caucasian populations, being also a marker of CVH changes and a predictor of future adverse events. On the other hand, renin and aldosterone were inversely related to CVH scores. In contrast, the counter-regulatory angiotensins [Ang (1-7) acting through Mas receptor, Ang (1-9) acting through Angiotensin Type 2 receptor, and alamandine] strengthen the beneficial properties of NPs. This evidence can be explained by both the effects on systemic hemodynamic and possible pleiotropic local functions regulating different pathways involved in the maintenance of CVH. Based on the current evidence, circulating levels of NT-proBNP, renin and aldosterone may affect CVH in apparently asymptomatic individuals and represent additional markers of residual cardiovascular risk.

## 1. Introduction

Cardiovascular diseases (CVDs) are the most common cause of mortality and morbidity worldwide.

The maintenance of a cardiovascular health (CVH) status represents an ideal condition to prevent or counteract the occurrence of CVDs. To this aim, a healthy lifestyle, including regular physical activity, a balanced diet, a normal body mass index (BMI), non-smoking status, normal values of blood pressure (BP), fasting plasma glucose and cholesterol levels, is needed and, in fact, it was included in the AHA Cardiovascular Health score (Life’s Simple 7 [LS7]) [1], subsequently updated to Life’s Simple 8 [LS8] to include sleep health [2]. A meta-analysis including 1,881,382 participants revealed a graded relationship between LS7 and the risk of CVDs [3]. A one-point increase in the ideal metric led to an 11–25% reduction of cardiovascular events (CVE). Intermediate CVH associated with a lower incidence of a composite endpoint [including myocardial infarction (MI), stroke, heart failure (HF), atrial fibrillation (AF), chronic kidney disease and peripheral artery disease] (−43%), MI (−55%), stroke (−35%), HF (−44%), and coronary artery disease (CAD) (−42%) [3]. Consistently, another study conducted in 210,443 subjects demonstrated that the achievement of a greater number of ideal or intermediate metrics was associated with 31–77% lower incidence of stroke, MI, HF and a composite variable of these events [4]. The LS8 has been recently associated with the lowest all-cause mortality and CVD mortality among individuals diagnosed with CVD [5], Despite the enrichment and refinement of LS8 compared to LS7, the predictive performance of the two scores is indistinguishable, raising some doubts on the value of a more complex score in the clinical practice [6].

Apart from the appropriate control of conventional cardiovascular risk factors, growing evidence suggests that higher level of cardiovascular protective hormones along with lower levels of unfavorable cardiovascular hormones significantly contribute to maintaining the CVH status and to preventing or delaying both CVDs and CVE occurrence. A previous investigation performed in a large community-based sample, the Framingham offspring study, assessed that the ideal CVH directly associated with a favorable profile of circulating cardiovascular biomarkers, including the amino terminal natriuretic peptides (NT-proNPs), and was inversely related to a less favorable cardiovascular hormonal profile including aldosterone levels [7]. Of note, the circulating NPs levels reported in the Framingham offspring study were within the normal range, not indicative of disease. Subsequent studies reinforced this evidence. Data from the Multi-Ethnic Study of Atherosclerosis (MESA) conducted in six centers across the USA (Baltimore, Maryland; Chicago, Illinois; Forsyth County, North Carolina; Los Angeles, California; New York, New York; and St Paul, Minnesota) showed that more favorable CVH scores were associated with lower concentrations of CVD-related biomarkers in both women and men, whereas NT-proBNP level showed a direct relationship with CVH scores [8]. Regarding the renin-angiotensin-aldosterone system (RAAS), the HyperPATH (International Hypertensive Pathotype) study reported that a higher LS7 score was associated with a lower level of serum and urinary aldosterone, lower plasma renin activity and a blunted increase in serum aldosterone upon Ang II infusion [9]. Moreover, the relevance of the RAAS was highlighted in a study performed in African Americans where aldosterone mediated the association of ideal CVH with incident type 2 diabetes mellitus through its effects on BP and glucose levels [10]. In the same cohort a higher attainment of ideal LS7 metrics was associated with lower serum aldosterone level [11].

The NPs and RAAS hormonal systems have been extensively investigated over the last decades [12,13]. These hormones exert opposite actions to maintain the hemodynamic and electrolyte-fluid balance. Moreover, through their complex biological actions, they play opposite effects on the cardiac and vascular remodeling process underlying the development of CVDs. The RAAS, through its main components renin, Ang II and aldosterone, may become, when excessive, an unfavorable hormonal player, whereas the components of the NPs family act as the hormonal protective determinants [13]. In addition, the alternative protein-enzymatic pathway of the RAAS, mediated by Ang II/Angiotensin type 2 receptor (Ang II/AT2R), Ang (1-7)/Mas receptor, Ang (1-9)/AT2R and alamandine, may support protective actions which are also synergistic to NPs [14].

The aim of this article is to review the available epidemiological evidence supporting the role of both NPs and RAAS hormonal systems as active contributors to CVH and informative biomarkers of CVH changes, including a discussion of the underlying molecular mechanisms. We will highlight the relevance of integrating cardiovascular hormone measurements into routine health assessments and making these innovations accessible to all for a comprehensive assessment of CVH status. Finally, we will address the novel concept that altered levels of these cardiovascular hormones and particularly of their balance, despite an appropriate control of conventional risk factors, may provide a new tool to identify residual cardiovascular risk and may support new preventive strategies in apparently asymptomatic individuals.

For our purposes we performed a literature search by using the following key terms: cardiovascular health, cardiovascular prevention, cardiometabolic risk, general population, natriuretic peptides, renin, angiotensin II, aldosterone, and Ang (1-7).

## 2. Experimental and Human Evidence Linking NPs to CVH

ANP, a major component of the NPs family, is mostly secreted by cardiomyocytes and is a potent natriuretic and diuretic hormone, due to both glomerular and tubular effects in the kidneys [13]. ANP increases the glomerular filtration rate and the filtration fraction by simultaneous dilation of afferent arterioles and constriction of efferent arterioles. It also inhibits water reabsorption though the renal cortical collecting duct and inhibits Na+ reabsorption by the renal inner medullary collecting duct [15]. Within the cardiovascular system ANP reduces BP through the reduced cardiac output mediated by both decreased preload and peripheral vascular resistance [16,17]. Furthermore, ANP reduces sympathetic tone by inhibiting arterial baroreceptor response and the release of catecholamines from autonomic nerve endings [18,19]. ANP directly reduces renin secretion, lowers circulating level of Ang II, and directly inhibits aldosterone synthesis from the glomerulosa cells of the adrenal cortex [20,21,22]. All described effects of ANP are mediated through the interaction with the type A guanylyl cyclase receptor (GCA). BNP, acting through GCA, like ANP, is a potent natriuretic, diuretic, vasorelaxant factor as well as an antagonist of the RAAS [23,24,25,26]. BNP is synthesized by the heart, and to a lesser extent by other organs [11]. Apart from their hemodynamic systemic effects, both ANP and BNP play several autocrine/paracrine functions within the cardiovascular system, such as anti-hypertrophic and anti-fibrotic effects. They also play a relevant lipolytic action and regulate BMI. At the cellular level, physiological concentrations of NPs favor cell viability, angiogenesis and proliferation, while opposing oxidative stress and inflammation. These effects likely result from an adequate control of the cellular metabolism including mitochondrial function. ANP was recently shown to act as a stimulator of autophagy, a complex intracellular process that delivers cytoplasmic constituents for degradation into lysosomes and is stimulated by environmental stress [27]. The activation of autophagy by ANP was revealed as a protective mechanism toward ischemia/reperfusion injury within the heart [28] and oxidative stress and inflammation in endothelial cells [29]. The third component of the family, CNP, has a modest natriuretic activity. It is secreted by endothelial cells, and it preserves endothelial function and structure in a paracrine manner. CNP plays potent anti-growth properties on vascular smooth muscle cells [30] and exerts vasorelaxant effects in the resistance vasculature to regulate BP [31,32]. Moreover, CNP contributes to maintaining coronary vasoreactivity [33], angiogenesis and remodeling. Similarly to ANP and BNP, CNP also acts as an antifibrotic factor within the heart. NPs are degraded by the type C natriuretic peptide receptor (NPRC) which is devoid of guanylyl cyclase activity. Neprilysin (NEP) also degrades NPs with a higher affinity for ANP [13].

Advanced age, kidney function, body mass composition, sex and ethnicity influence the circulating level of NPs [13]. Levels are higher in white individuals, in females and older subjects. The age-related changes in plasma ANP and BNP levels are influenced by the age-dependent decrease in left ventricular stroke volume and left ventricular volumes along with an increase in left ventricular mass, left ventricular and arterial elastance in both sexes [34,35]. It is likely that increasing intramural pressure, myocardial inflammation and fibrosis mediate the impact on NPs level in advanced age.

### 2.1. Evidence Supporting the Cardiovascular and Cardiometabolic Protection Exerted by NPs

**Figure 1:**
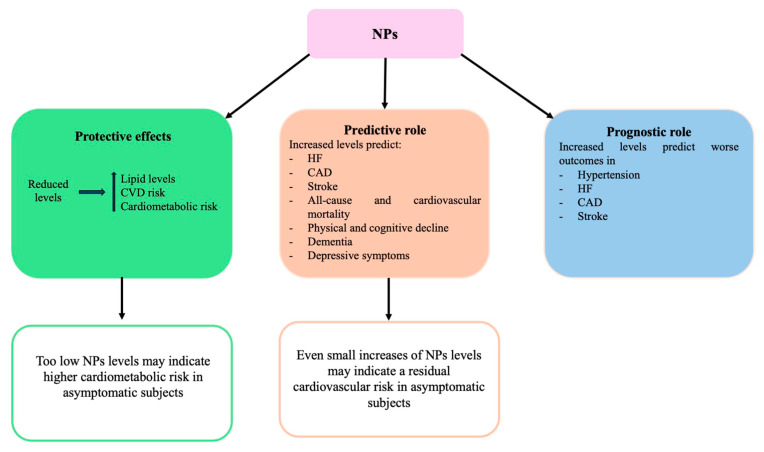
Protective and predictive effects of NPs toward cardiovascular and cardiometabolic risk in the general population. The prognostic role exerted in several major CVDs is also shown.

The role of NPs in maintaining a healthy circulation and metabolism explains the benefits deriving from their regular synthesis and normal circulating concentrations. As a proof of their protective effect, it has been repeatedly reported that lower ANP and BNP levels are associated with increased cardiometabolic risk in different populations [36]. As an interesting example, the black race shows lower NPs levels because of an epigenetic differential modulation, as compared to the white race [37,38], and a higher cardiometabolic risk profile [39]. Previous work in community participants free of heart disease showed that low plasma concentrations of ANP and BNP at midlife are associated with increases in CVDs and impaired metabolic health [40,41]. A recent study performed in healthy 50-year-old adults reported that both ANP and BNP were negatively related to lipid levels and other markers of the metabolic syndrome, and they reduced the incidence of impaired cardiovascular health over 15 years of observation [42]. It can be speculated that “too low” NPs levels, as marker of a NP deficiency state, may indicate higher cardiometabolic risk in asymptomatic subjects (Figure 1).

### 2.2. Evidence Supporting the Role of NPs in Cardiovascular Risk Prediction Within General Populations

The protective effects of NP have other important implications (Figure 1). In fact, NPs levels increase as soon as an injury occurs to counteract the damage and oppose the development of disease. Therefore, elevated NPs levels have a negative prognostic value in different clinical conditions. Increased NT-proBNP levels indicate a higher risk of cardiovascular and all-cause mortality in hypertensive patients with stage 1 hypertension. Higher NPs levels predict recurrent MI and angina in CAD patients. A similar negative prognostic role of NPs holds true in HF [13]. A therapeutic strategy able to lower BNP or NT-proBNP level may indicate favorable reversed LV remodeling and improved ventricular function in pre-HF patients, as recently shown in a study exploring the optimal first-line antihypertensive drugs [43].

Regarding the general population, a study including more than 90,000 individuals from all over the world (the NPs studies collaboration), revealed that NT-proBNP level (median concentration; 64 pg/mL) predicted first-onset HF and augmented CAD and stroke occurrence, suggesting that NT-proBNP concentration assessment could be used to integrate HF into CVD primary prevention [44]. A recent large analysis including 164,054 individuals from the general population evaluated the predictive value of known cardiovascular biomarkers (NT-proBNP, BNP, C-reactive protein, cardiac troponin I, cardiac troponin T) [45]. It was observed that the cumulative incidence of atherosclerotic events gradually increased with increasing biomarker concentrations (median concentrations of 43.8 ng/L for NT-proBNP, 14.9 ng/L for BNP). All considered biomarkers were associated with all-cause mortality, HF, stroke and MI. The addition of these biomarkers to a model including established risk factors such as age, total cholesterol, HDL cholesterol, smoking status, diabetes, systolic BP, and self-reported use of antihypertensive drugs, improved the C statistic (from 0.812 [95% CI, 0.8021–0.8208] to 0.8194 [95% CI, 0.8089–0.8277]) for the prediction of CVE, particularly HF and all-cause mortality, and the classification of cardiovascular risk. The incremental value of biomarkers was greater in people aged 65 years or older. The magnitude of change in the C statistic for all-cause mortality and HF was comparable to that achieved with the addition of coronary calcium scoring to classic cardiovascular risk factors. Notably, the association between the incidence of CVE and levels of NT-proBNP was significant (hazard ratio [HR] 1.14; 95% confidence interval [CI], 1.08–1.22], similarly to what was observed for BNP (HR 1.14; 95%CI, 1.12–1.16) [45]. In addition, the highest HR was observed for NT-proBNP for all outcomes except for incident MI, with increasing predictive value of NT-proBNP in older people. The incremental value of NPs was maintained over a 10-year period [45]. Another recent study has shown that, out of forty-eight proteins, forty-three metabolites, age, sex and systolic blood pressure levels, BNP level was most consistently associated with the risk of imminent MI in a community of individuals without prior CVD from six European cohorts [46].

The tight relationship between NPs and BMI has been underscored in a recent investigation on the association between NT-proBNP and all-cause mortality and cardiovascular mortality in individuals with different BMIs, including obese individuals. The predictive role of NT-proBNP was also confirmed in this context, and a significant correlation with cardiovascular mortality was found only for NT-proBNP level ≥ 300 pg/mL and only among individuals with abdominal obesity [47].

In a community based cohort of 8938 adult subjects with diabetes and prediabetes, the addition of NPs to the WATCH-DM risk Score, which includes 10 clinical, laboratory, and ECG variables (age, BMI, systolic BP, diastolic BP, serum creatinine, fasting plasma glucose, high-density lipoprotein cholesterol, QRS duration, history of MI, history of coronary artery bypass graft) improved the prediction of HF incidence [48]. Elevated NPs level (NT-proBNP ≥ 125 pg/mL or BNP ≥ 40 pg/mL for BMI ≤ 30 kg/m^2^; NT-proBNP ≥100 pg/mL or BNP ≥30 pg/mL for BMI > 30 kg/m^2^) demonstrated better risk stratification among individuals with low/intermediate vs. high WATCH-DM(i) scores. Among participants with low/intermediate WATCH-DM(i) scores, the incidence of HF was six-fold higher in subjects with elevated NPs level (8.2 vs. 1.4%). Among participants with high WATCH-DM(i), there was only a three-fold gradient in HF incidence across the NP level strata (4.7% vs. 14.1% in low vs. high NP groups) [48].

Based on the evidence provided by the studies reported above, NPs might be considered as a very sensitive “alarm system”, as shown in a cohort of non-HF patients from the Essen Coronary Artery Disease registry [49]. In this latter study, very modest increases of NPs predicted mortality. In details, BNP levels > 9.6 pg/mL in men and >29 pg/mL in women, and NT-proBNP thresholds of 65 and 77 pg/mL for men and women, respectively, were associated with a 2.5-fold increase in all-cause mortality, independently from the coexistence of hypertension, CAD and higher BMI [49,50]. Of note, the reported levels of NPs in this study were not indicative of disease. This result supports the role of NPs as early predictors of cardiovascular damage and key factors to preserve CVH.

Interestingly, it should be mentioned that a study evaluating organ aging using plasma proteomics data, predicting diseases and aging effects, found that NT-proBNP and cardiac troponin T had the strongest weight in the heart aging model also suggesting a tight connection between subclinical heart disease and the ‘normal’ heart aging process [51].

Other investigations underscored the role of NPs as sensitive markers of early abnormalities of the health status. Notably, a study evaluating physical capability, a key component of healthy aging, in an old British cohort of men and women found that a higher level of NT-proBNP could identify those in midlife at risk of accelerated physical decline [52]. The elevated NT-proBNP level predicted worsening performance on activities of daily living and cognitive decline [53]. Higher NT-proBNP level associated with incident disability in older adults [54]. Consistently, it has been reported that NT-proBNP level may be directly related to age-dependent structural and functional brain changes, including decline in brain tissue volume, cognitive impairment, and increased depressive symptoms [55]. In a study by Ostovaneh et al. [56] baseline NT-proBNP level was associated with the future development of impaired cognitive function. Most importantly, a 3-year increase of NT-proBNP level over time was associated with an increased risk of future dementia whereas a decrease of NT-proBNP level was associated with reduced risk of dementia (Figure 1).

All above discussed evidence supports the view of NPs as an integrated measure of cumulative exposures to relevant stressors across life, as a suitable biomarker to capture early end-organ damage and even as an aging biomarker. Furthermore, it may be speculated that, in the presence of an effective control of conventional cardiovascular risk factors, even small increases in NPs levels may detect a residual cardiovascular risk in apparently asymptomatic individuals (Table 1), (Figure 1).

### 2.3. Therapeutic Implications of NPs in CVDs

Despite several efforts to develop NP-based therapeutic strategies, very few therapeutic interventions based on the cardiovascular beneficial properties of NPs are nowadays available [13]. Following several attempts with synthetic analogues and NEP inhibitors, the recent introduction of Angiotensin Receptor Neprilysin inhibitors (ARNi) has revealed success in their systemic hemodynamic and cellular local effects, mainly through the increase of circulating ANP. This drug has currently indication only for the treatment of HF with reduced systolic function, HFrEF [57,58], although it also acts as an efficacious antihypertensive agent. We demonstrated that increased ANP levels consequent to NEP inhibition can preserve mitochondrial function and the autophagy/mitophagy process in HFrEF patients, contributing to the improvement of left ventricular systolic function [59]. In the context of hypertension, we and others demonstrated that ARNi protects from the target organ damage development by both its antihypertensive action and local effects [13,60,61]. Among the NP synthetic peptides, the mutant ANP (MANP) has been recently introduced into the clinical arena by carrying pilot clinical trials in hypertensive patients without and with cardiometabolic syndrome. These studies revealed that MANP is an efficacious blood pressure-lowering agent and reduces the cardiometabolic risk [62,63], further supporting the beneficial effects of the native ANP (Table 2).

## 3. The Implications of Both Classical and Counter-Regulatory RAAS in CVH Maintenance

The RAAS regulates BP through its sodium/water retaining effect and vasoconstriction [12]. Renin is a highly specific endopeptidase produced in the juxtaglomerular cells of the kidney and represents the rate limiting enzyme of the RAAS. It generates angiotensin I (Ang I) from the cleavage of angiotensinogen (AGT). Ang I, in turn, is the substrate for angiotensin converting enzyme (ACE) (a kininase II enzyme) to generate Ang II [64]. The latter peptide binds to the angiotensin type II receptor (AT1R) to promote vasoconstriction and to stimulate aldosterone secretion from the adrenal gland. The classical view of the RAAS includes prorenin/renin/AGT/Ang I/ACE/Ang II/AT1R. Another receptor, AT2R, plays opposing effects to those exerted by AT1R. In fact, the AT1R mediates vasoconstriction, thirst, release of vasopressin and aldosterone, renal sodium reabsorption, hypertrophy, proliferation and fibrosis, inflammation, angiogenesis, vascular aging, and atherosclerosis [64,65,66]. On the other hand, the AT2R mediates vasodilation, antiproliferative, antihypertrophic, antifibrotic, and antithrombotic effects [67,68,69,70], Importantly, a crosstalk between the two Ang II receptors has been documented [71,72,73].

Of note, renin was revealed as an important protein in kidney aging in the above-mentioned study evaluating organ aging using plasma proteomics data [51].

The RAAS is a pathway mediated by multiple enzymatic reactions that produce a wide variety of peptides. We currently recognize the existence of several functional angiotensins: Ang II, Ang III, Ang IV, Ang-(1-7), Ang-(1-9), alamandine. The Ang (1-7), obtained by the cleavage of Ang II through ACE2, interacts with the Mas receptor to preserve endothelial function and mediate vasodilation; it exerts antihypertrophic, antifibrotic, anti-inflammatory and antithrombotic properties [74,75,76,77,78,79]. Therefore, the ACE2/Ang-(1-7)/Mas receptor supports the protective functions exerted by the Ang II/AT2R interaction. It has been reported that the ACE2/Ang (1-7) axis modulates the immune response, influencing the microbiota composition, thus contributing to CVDs also through modulation of metabolic parameters, such as weight, adiposity and lipid profile [80].

Ang (1-9), acting through AT2R, and alamandine, acting through MrgD, also exert beneficial cardiovascular properties such as decreased cardiac fibrosis, decreased myocardial hypertrophy, vasodilation, decreased BP, natriuresis, and NO synthesis [14,81,82,83]. Another key component of the system is the prorenin receptor that binds prorenin and activates it in a non-proteolytic manner to allow the renin formation and to increase local Ang II production as well as initiate Ang II-independent signaling [84].

**Figure 2:**
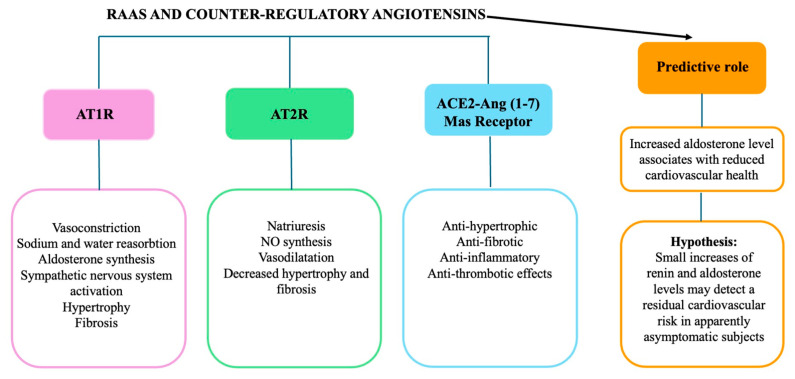
Main cardiovascular effects of RAAS and of counter regulatory angiotensins. Based on the epidemiological evidence of increased aldosterone level in association with reduced CVH, we propose that they may serve as marker of residual cardiovascular risk.

As far as we can tell today based on the above-mentioned evidence, the net biological functions of the RAAS results from the balance between the arms of ACE/Ang II/AT1R/AT2R and that of the counter-regulatory angiotensins and their receptors [85] (Figure 2).

The overactivation of RAAS promotes dangerous effects through the AT1R. In this regard, the evidence provided by RAAS blockade-based therapies has largely grown over the years and supports this concept. The blockade of Ang II can be achieved by both the ACE inhibition (ACEi) and the AT1R antagonism with specific molecules, collectively defined as Angiotensin Receptor Blockers (ARBs) or Sartan compounds. The blockade of the mineralocorticoid receptor, and more recently the direct inhibition of aldosterone synthesis, represent additional strategies to inhibit the aldosterone pathway within the RAAS [86]. We learned about the protective effects of RAAS inhibition in all major CVDs, such as hypertension and ischemic heart disease, HF [12]. At the vascular level, RAAS inhibition reduces arterial stiffness and might prevent the development of cognitive impairment [87]. At the cellular level, RAAS-blockade improves mitochondrial function by downregulating the mammalian target of rapamicyn (mTOR) and growth hormone/insulin growth factor (IGF)-1 signaling, stimulating AMP-activated protein kinase (AMPK) and sirtuins activity [88,89]. More recently, it has been proposed that RAAS blockers might inhibit the epigenetic transcription of hypertrophy-related genes [90]. Consistently, different studies have shown that RAAS blockers exert an anti-inflammatory role by reducing cytokine production, expression of adhesion molecules and plasma C-reactive protein, these actions contributing to the protective effects against cardiac and vascular damage as well as the aging process [91]. The activation of AT2R represents an additional interesting tool to achieve cardiovascular protection [92]. AT2R has been detected in the inner mitochondrial membrane and its activation has been demonstrated to stimulate NO production [93].

The recent knowledge on the beneficial effects of ACE2, Ang-(1-7), Ang-(1-9) and alamandine has raised some interest on evaluating their effects toward the cardiovascular risk. Indeed, Ang-(1-7)-mediated Mas receptor activation has been shown to contribute to the favorable effects of AT1R antagonism on NO bioavailability and microvascular remodeling, independently of AT2R activation and BP control [94]. In addition, the effect of Ang-(1-7) on sympathetic nervous system activity to restore β2 vascular adrenergic receptor signaling and reduce cardiovascular risk during aging is currently being evaluated in ongoing clinical trials [81] (Table 2).

## 4. Perspectives

**Figure 3:**
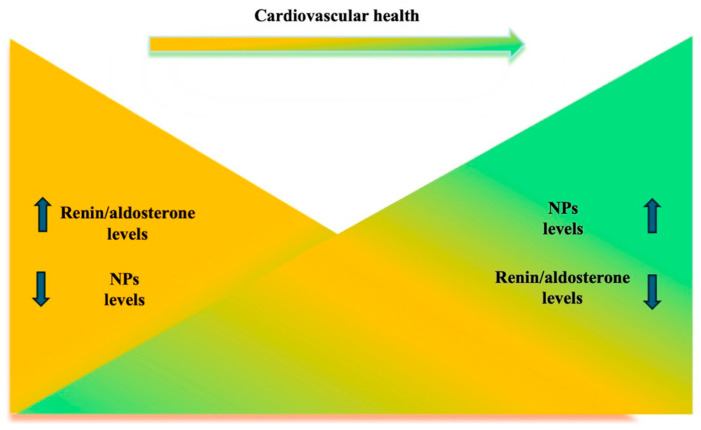
Several factors are involved in the maintenance of CVH. Current evidence supports the view that a decrease of both renin and aldosterone levels and an increase of NPs levels behave as key contributors to CVH (left side). Altered levels of these hormones, despite an appropriate control of conventional risk factors, may indicate a residual cardiovascular risk and the need of more intensive preventive strategies in apparently asymptomatic individuals.

Because of their opposing biological effects, the balance between NPs and RAAS hormonal systems may play an important role to preserve ideal CVH in people (Figure 3).

Based on the epidemiological evidence, increased NPs levels and reduced renin and aldosterone levels may be considered as key determinants of CVH. Therefore, “abnormal” hormone levels or alteration in their balance may represent useful biomarkers to predict CVH changes and identify apparently asymptomatic subjects at higher cardiovascular and cardiometabolic risk. To test this hypothesis many studies have been carried out particularly investigating NPs levels, more often than renin and aldosterone levels, in several communities and the results always supported the NPs predictive value. However, despite the great potential offered by integrating these cardiovascular hormone measurements into routine health assessments, several difficulties still limit their introduction into cardiovascular preventive strategies and there are no specific guidelines on introducing these biomarkers into routine tests. A main limitation is represented by the variability of cut-off levels reported among the different studies, mainly related to age, sex, BMI and ethnicity. An agreement to define the “normal” range of NPs levels in the community, eventually adjusted based on the major variables, is urgently needed to better recognize an increased cardiovascular risk in apparently healthy individuals. This complex issue has been intensively investigated regarding NT-proBNP level and has led to the conclusion that if the assessment of NT-proBNP level is introduced in the clinical screening in the general population, interpretation of NT-proBNP levels will require that adjustments for age and gender are used to identify patients at potentially higher cardiovascular risk [95]. Furthermore, to define the threshold of NP deficiency and the consequent increased cardiometabolic risk, a great effort has been made to establish the reference range for NT-proBNP across the lifespan of a healthy US population accounting for age, sex and ethnicity [96]. However, the authors of this work chose an arbitrary value to achieve their goal. Therefore, further work is required to better assess the criteria and appropriately use NPs levels to identify at risk individuals [97].

Another relevant issue is the need to select specific and sensitive assay methodologies able to assure reproducible results in all individuals. In addition, the cost-benefit ratio has to be taken into account, particularly when considering that the measurement of these hormone levels should be made accessible to all subjects undergoing preventive screenings (at workplaces, sport centers, etc.) with the aim of properly identifying subjects at increased risk on time.

Finally, clear evidence should be provided about the ability of these hormonal levels, once added to the current risk-estimation models that are based on the conventional cardiovascular risk factors [98], to significantly improve risk prediction.

These limitations should be overcome in the future years, so that the measurements of either “too low” or “too high” NPs levels, as well as of higher renin and aldosterone levels, may become more accessible and affordable to ensure broader public health benefits. In the presence of an adequate control of conventional modifiable cardiovascular risk factors, this approach may provide useful indications to characterize residual cardiovascular risk and to design more specific preventive strategies in apparently healthy individuals.

## 5. Study Limitations

This article was prepared as a narrative and not as a systematic type of review.

Whereas the literature offers abundant evidence on the implications of NT-proBNP in CVH, no large evidence is available with regard to ANP and CNP limiting the discussion on the contribution of the whole NPs family to the maintenance of CVH. However, we know that the components of the NPs family exert similar functions in the cardiovascular system.

Similarly, with regard to the RAAS, the renin and angiotensin II levels are not commonly assessed, and we based our discussion mainly on the evidence provided by studies assessing the aldosterone level.

Furthermore, most of the investigations reported in the literature were performed in males. Finally, Caucasian populations were considered in the majority of the studies with limited evidence in African and Asian populations.

## Figures and Tables

**Table 1:** Main implications of NPs in cardiovascular health.

Measure of cumulative exposure to relevant stressors across life
Biomarker able to capture early end-organ damage in apparently asymptomatic individuals
Aging biomarker, including the prediction of cognitive decline and dementia

**Table 2:** Therapeutic strategies targeting NPs and RAAS to modulate their cardiovascular effects and to enhance cardiovascular health.

Inhibition of: ACE AT1R RENIN ALDOSTERONE NEP
Activation of: AT2R ACE2 Ang (1-7)/Mas receptor Ang (1-9) Alamandine ANP, BNP, CNP, NPRA
Peptides analogs: MANP

Legend: ACE = angiotensin converting enzyme; ACE2 = angiotensin converting enzyme 2; AT1R = type 1 angiotensin receptor; AT2R = type 2 angiotensin receptor; NEP = neprilysin; ANP = atrial natriuretici peptide; BNP = brain natriuretic peptide; CNP = C-type natriuretic peptide; NPRA = type A natriuretic peptide receptor; MANP = mutant ANP.
